# Biosynthesis of Novel Ag-Cu Bimetallic Nanoparticles from Leaf Extract of *Salvia officinalis* and Their Antibacterial Activity

**DOI:** 10.3390/life13030653

**Published:** 2023-02-27

**Authors:** Maqsood Ahmad Malik, Shroog ShdiedRoyji Albeladi, Saleh Mohammed Al-Maaqar, Abdulmohsen Ali Alshehri, Shaeel Ahmed Al-Thabaiti, Imran Khan, Majid Rasool Kamli

**Affiliations:** 1 Chemistry Department, Faculty of Science, King Abdulaziz University, P.O. Box 80203, Jeddah 21589, Saudi Arabia; 2 Department of Biological Sciences, Faculty of Science, King Abdulaziz University, P.O. Box 80203, Jeddah 21589, Saudi Arabia; 3 Applied Science Section, Faculty of Engineering and Technology, Aligarh Muslim University, Aligarh 202002, India; 4 Center of Excellence in Bionanoscience Research, King Abdulaziz University, P.O. Box 80203, Jeddah 21589, Saudi Arabia

**Keywords:** biosynthesis, phytochemicals, reducing agents, stabilizing agents, biofilm, antibacterial agents

## Abstract

Bimetallic nanoparticles exhibit bifunctional or synergistic effects prevailing between two metals with the capabilities of enhanced electronic, catalytic, and optical properties. Green synthetic routes have gained tremendous interest because of the noninvolvement of toxic and harmful chemical reagents in preparation. Therefore, we develop bimetallic Ag-Cu nanoparticles (Ag-Cu NPs) through an eco-friendly and biocompatible preparation method. In this study, Ag-Cu NPs have been synthesized from leaf extracts of the commonly known sage, *S*. officinalis. The extract has a rich phytochemical composition, including bioreducing polyphenols, flavonoids, and capping/stabilizing agents. An array of well-known spectroscopic and microscopic techniques were used to characterize the as-prepared Ag-Cu bimetallic nanoparticles, including X-ray diffraction (XRD), ultraviolet-visible spectroscopy, Fourier transform infrared spectroscopy (FTIR), scanning electron microscopy, transmission electron microscopy, and energy-dispersive X-ray spectroscopy. The size of the Ag-Cu NPs was found to be 50 nm with a spherical shape and an almost uniform distribution. The antibacterial effect was further evaluated using agar well diffusion and disc diffusion assays. Ag-Cu NPs exhibit antibacterial and antibiofilm properties against Gram-positive and Gram-negative bacteria strains. The minimum inhibitory concentration (MIC) of Ag-Cu NPs was between 5 g/mL and 15 g/mL. The Ag-Cu NPs inhibit biofilm formation at 25 g/mL and 50 g/mL. The results of biogenic Ag-Cu NPs provide novel antibacterial activity against Gram-positive and Gram-negative bacteria, as well as antibiofilm activity. Hence, Ag-Cu NPs might serve as a novel antibacterial agent with potential antibacterial and antibiofilm properties.

## 1. Introduction

Bimetallic nanoparticles possess far greater properties than their constituent elements. In various fields, including catalysis, electrocatalysis, and magnetism, the combination of noble metals and first-row transition metals is attracting increasing interest. Therefore, bimetallic nanocrystals composed of noble and non-noble metals are of great interest. Bimetallic nanocrystals can change significantly in their chemical, mechanical, electrical, magnetic, and other properties due to the presence of the two metals and their interactions [1]. Nanoobjects have attracted researchers’ attention for years, and bimetallic nanoparticles are no exception [2]. Thus, two metals alloying leads to atoms being isolated at particular sites. The presence of particles close by also induces a change in the electronic structure of the resulting alloy [3]. The anticipated benefits of the material pertain to its chemical and physical properties, including catalytic, magnetic, photophysical, and electronic properties [4,5,6,7,8,9,10].

In recent years, bimetallic nanoparticles have garnered much attention in scientific and technological communities because of their superior physicochemical properties compared to monometallic nanoparticles [11]. Compared to their monometallic counterparts, bimetallic nanoparticles display unusually improved optical, catalytic, and electrical capabilities [12,13]. This is because bimetallic nanoparticles can perform dual functions or have synergistic effects. The preparation process frequently decides the distribution and origin of the metals, which can result in core–shell structures, alloy structures, random alloy structures, clusters within the cluster, and subclusters [14]. Additionally, the size of the particles affects the alloying or phase aggregation of bimetallic and trimetallic nanoparticles [15,16]. Many other types of bimetallic nanoparticles, such as Ag-Ni [17], Ag@Fe [18], Ag@MnO_2_ [19], Co@Ag [20], Ag/Au [21], Ag/Pt [22], and Au/Pd [23], have recently been the subjects of research.

The high surface area-to-volume ratio of silver nanoparticles is attracting much attention due to their increased pharmacological efficacy in treating microbial pathogens. Additionally, they are thought to have anti-inflammatory and antiangiogenic properties [24,25,26,27,28,29]. The ease of availability, low cost, and similar characteristics of copper nanoparticles make them enormously interesting [30,31]. Copper nanoparticles offer many valuable applications, including in heat transfer systems and fuel and solar cells, and they display electrical, catalytic, antifungal, and antibacterial properties [32,33,34,35,36,37,38,39].

Nanomedicine is a growing field that has already made much progress and has the potential to reach important milestones in a wide range of clinical applications. However, there are still barriers that make it hard to put nanomedicine into practice [40,41]. Recent studies have shown that nanomedicines can be effective in various medical contexts, from disease diagnosis to therapy, inspiring optimism for their future. Nanotechnology developments have revolutionized the health care system in this age of nanoscience by developing nanostructures that greatly enhance the diagnosis and therapeutic aspects of many diseases [42].

Antibiotic resistance development is a major problem associated with widespread antibiotic use [43]. Treatment of various bacterial illnesses is frequently hampered by antibiotic resistance [44,45]. Moreover, biofilm formation in bacterial infections, which has been indicated to be the most challenging element to address [46], further amplifies the likelihood of medication resistance. In addition to the need for new antibiotics, there is an urgent need for novel tactics that target the survivability of infections. Our research centered on the biosynthesis of non-toxic and non-polluting Ag-Cu NPs as a straightforward method for producing Ag-Cu NPs from *S. officinalis* extract. Biosynthesized Ag-Cu NPs were used to combat four pathogenic bacterial strains as antibacterial and antibiofilm agents.

## 2. Materials and Methods

### 2.1. Materials

All the chemicals utilized throughout the investigation were of extremely high-purity and analytical grade, and did not undergo any additional purification steps. Silver nitrate (AgNO_3_) (purity-99.0%), Copper(II) nitrate trihydrate (Cu(NO_3_)_2_·3H_2_O) (purity-99.99%), and ethanol (purity-95.00%) were purchased from Sigma Aldrich. The stock solutions of (AgNO_3_ = 0.01 mol. dm^−3^) and (Cu(NO_3_)_2_ = 0.01 mol. dm^−3^) were prepared in double distilled water and then stored in a dark environment for further use. Our investigation utilized plant material from the local market in Jeddah, which is located in the Kingdom of Saudi Arabia.

### 2.2. Synthesis of Ag-Cu Bimetallic Nanoparticles

In order to thoroughly clean the sage leaves, tap water was first used to rinse dirt from the plant’s surface, followed by ultrapure water. After the leaves were cleaned, they were dried in an environment that was clean, dry, and free of contaminants. Next, the dried leaves were milled into a fine powder using a grinder, and then 10 g of this powder was dispersed with constant stirring in 200 mL of deionized water and heated at 60 °C for 2 h. Afterward, upon vacuum filtration of the leaf extract, the filtered solution thus obtained was kept in a dark place to cool by 4 °C before being used.

Under ideal experimental conditions, Ag-Cu NPs were prepared using a simple one-pot biosynthetic approach in which metal salts of AgNO_3_ and Cu(NO_3_)_2_ were constantly stirred together at room temperature in a 250 mL beaker. To this reaction mixture of metal salts, 15 mL of aqueous *S. officinalis* leaf extract was added under constant stirring. The biosynthesis of Ag-Cu bimetallic nanoparticles is confirmed by the physically observed color change in the experimental solutions, where the color changes from light brown to dark brown. After that, the so-obtained dark brown reaction mixture was centrifuged at 5000 rpm for 20 min to separate the reaction precipitate. Furthermore, the acquired solid material was thoroughly washed several times with deionized water and ethanol, followed by drying of the solid material at 120 °C for 12 h.

### 2.3. Characterization of Ag-Cu Bimetallic Nanoparticles

Bimetallic Ag-Cu nanoparticles were initially analyzed by using the UV-visible spectroscopy technique. The analysis was attained using a spectrophotometer (UV-2450, Shimadzu, Kyoto, Japan), and the UV-visible analysis of all sample spectra was conducted at room temperature. In addition, the functional group analysis was performed using FTIR with a Bruker instrument with a resolution of 4 cm^−1^ at room temperature. The investigation was used to confirm the possible role of reduction/stabilization attained by the phytochemical composition of *S. officinalis* leaf extract in the biogenesis of Ag-Cu bimetallic nanoparticles. Furthermore, the crystalline morphology of biosynthesized Ag-Cu nanoparticles was analyzed via X-ray powder diffraction (XRD) examination. The analysis was determined using a Bruker X-ray diffractometer (angle 2θ, within range of 20–80) operated at a voltage of 40 kV with a current of 30 mA. Using a Bruker Raman microscope, the Raman spectrum of the synthesized material was recorded in the spectral region of 50 to 2000 cm^−1^.

Meanwhile, the surface morphology of biosynthesized Ag-Cu nanoparticles was determined using SEM analysis. The energy-dispersive X-ray spectroscopy (EDX) was performed with a TECHNAI-320 KV JAPAN operated at 80 kV. Furthermore, the elemental composition of the biosynthesized Ag-Cu bimetallic nanoparticles was analyzed using a QUANTA FEG 450. In addition, transmission electron microscopy (TEM) analyses at 200 kV further described the size, shape, and morphology associated with biosynthesized bimetallic Ag-Cu nanoparticles.

### 2.4. Bacterial Strain and Growth Conditions

In this study, *Klebsiella pneumoniae*, *Escherichia coli*, *Staphylococcus aureus*, and *Staphylococcus epidermidis* were used to determine the antimicrobial activity of the Ag-Cu NPs. All the strains were obtained from the Microbiology Laboratory, Department of Biology, King Abdulaziz University, Jeddah, Kingdom of Saudi Arabia. All the bacterial strains were cultured in nutrient broth overnight and were stored at −80 °C in NB broth with 24% glycerol.

### 2.5. Minimum Inhibition Concentration (MIC)

Microbroth dilution assays were performed per CLSI protocols to determine the minimum inhibitory concentration (MIC). Ag-Cu NPs were dissolved into 1% dimethyl sulfoxide to provide a stock solution (100 g/mL) (DMSO; Sigma-Aldrich, Inc., St. Louis, MO, USA). The MIC for the Ag-Cu NPs was determined by measuring the lowest concentration at which growth inhibition was detectable. In brief, a 96-well plate was used for MIC and was incubated for 24 h at 37 °C. After incubation, 5 μL of Resazurin sodium salt dye solution (R7017 Sigma-Aldrich) was added to each well. Ampicillin was used as a positive control and 1% DMSO as a negative control.

### 2.6. Agar Well Diffusion Assay

The antibacterial activity was measured using the agar well diffusion method. Briefly, 10^6^ colony-forming units per milliliter of Mueller–Hinton agar medim (MHA) was inoculated with the bacterial strains. Five holes were punched into each culture plate using a sterilized cork borer. As a positive control, 100 μL of amoxicillin was employed, while sterile distilled water (SDW) served as a negative control. The remaining three holes each received 100 μL of nanoparticle samples with concentrations of 25, 50, and 75 μg/mL. The plates were incubated for 24 h at 37 °C. The zone of inhibition that emerged around the wells was reported as the mean standard deviation (SD) of experiments performed in triplicate.

### 2.7. Disk Diffusion Assay

Disk diffusion assay further checked the antibacterial activity of synthesized Ag-Cu NPs. First, Mueller–Hinton agar medium (MHA) was inoculated with the bacterial strains (10^6^ colony-forming units/mL). Then, a 7 mm paper filter disc impregnated with different concentrations of nanoparticles (25, 50, and 75 μg/mL) was placed on the agar, and the Ag-Cu NPs were allowed to diffuse into the medium for 30 min at room temperature. Oxacillin was used as a positive control, and SDW was used as a negative control. Finally, the plates were incubated at 37 °C for 24 h. The zone of inhibition was recorded as the mean ± standard deviation (SD) of triplicate experiments.

### 2.8. Biofilm Inhibition Assay

The crystal violet technique with a 96-well flat-bottomed microtiter plate was used to verify the antibiofilm property of Ag-Cu NPs. Bacterial strains were grown in nutrient broth supplemented with either 25 g/mL or 50 g/mL of Ag-Cu NPs at 30 °C for 24 h. Microplates were washed twice with phosphate-buffered saline (pH 7.4) to remove cells after incubation. Once 15 min had passed, crystal violet solution (0.1%, 200 L) was used to stain the biofilm. After that, ethanol (95%, 200 L) was used to clean the wells twice before PBS was used again. This biofilm production was measured quantitatively at OD470 nm with an iMark microplate reader (Bio-Rad Laboratories, ArIrvine, CA, USA).

## 3. Result and Discussions

**Figure 1:**
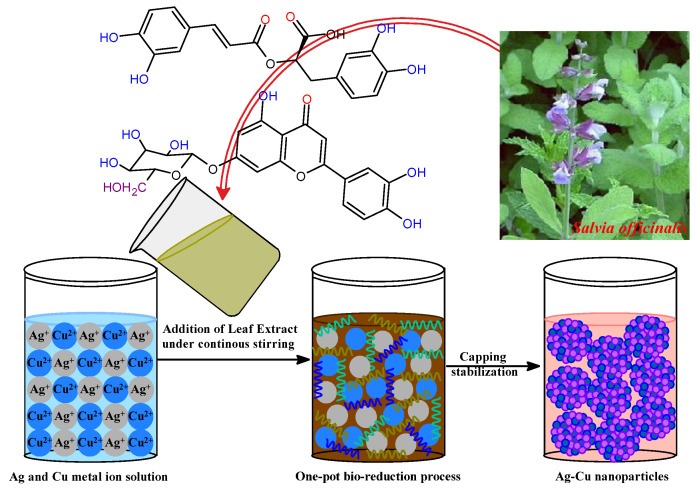
Possible mechanism of bimetallic Ag-Cu nanoparticle formation.

*Salvia officinalis*, a medicinal shrub of the family Lamiaceae [47,48], possesses many therapeutic activities, including antibacterial, antioxidant, anticancer, hypoglycemic, and anti-inflammatory actions [49,50]. A long history exists of *S. officinalis*, and extracts of its roots, leaves, and stems are reported with their various therapeutic activities in traditional medicine, with numerous pharmaceutical purposes [45,51,52]. These active phytochemicals include saponins, flavonoid glycosides, and phenolic compounds such as coumarins, tannins, glucosides, flavonoids, steroids, and terpenes, in addition to some multi-functional proteins [53,54,55,56]. *S. officinalis* has several phytochemicals in its aqueous extract. These phytochemicals have antioxidant activities and can combine Ag^+^ and Cu^2+^ ions into Ag-Cu nanoparticles. In addition, phytochemicals were responsible for forming numerous coordinate interactions between metal ions and the nanoparticles as they were being stabilized upon the biogenesis of bimetallic Ag-Cu nanoparticles. Because of this, the biocompatible aqueous extract of *S. officinalis*, being a rich source of phytochemicals, played its role as a bioreducing and stabilizing agent in the biosynthesis ecofriendly bimetallic Ag-Cu nanoalloy. During this bioreduction process, the Ag-Cu nanoalloy was yielded upon continuous stirring at a temperature of 50 °C for half an hour. Figure 1 presents a schematic representation of how the development of an Ag-Cu nanoalloy could be characterized. In the present study, the biofabrication process was attained after the aromatic phenolic –OH groups of *S. officinalis* extract served as reducing agents. Also, possible (NH)C=O groups of phytocomposition can be adsorbed onto the surface of Ag-Cu metal atoms, responsive after electronic conduction, and as such, are behind the biosynthesis of Ag-Cu clusters. In addition, functional groups, such as –C=C and –C=O, are responsible for stable surface passivation and serve as stabilizers to overcome aggregation of Ag-Cu nanoclusters. Phytochemically stabilized Ag-Cu NPs were subjected to additional characterization by various spectroscopic techniques before being utilized as photocatalytic and antibacterial agents against a selection of pathogens. The synthesized Ag-Cu NPs stabilized by phytochemicals can be used for anticancer and antibacterial actions.

### 3.1. UV-Visible and FTIR Spectral Analysis of Ag-Cu NPs

**Figure 2:**
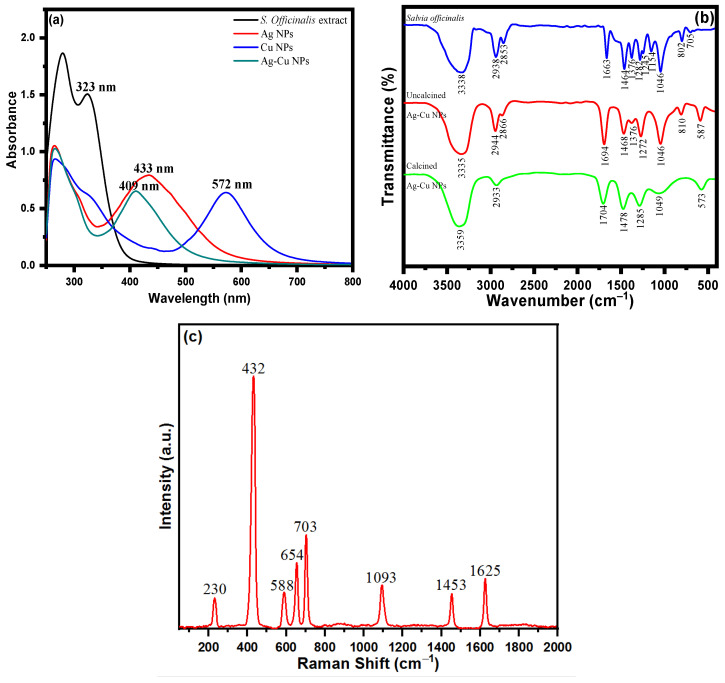
(**a**) UV-visible spectra of *S. officinalis* aqueous extract, Ag NPs, Cu NPs, and bimetallic Ag-Cu NPs; (**b**) FTIR spectra of *S. officinalis* aqueous extract and uncalcined and calcined Ag-Cu NPs; and (**c**) Raman spectrum of Ag-Cu NPs.

UV spectroscopy was used to analyze the surface phenomena and functionalization of nanoparticles. The UV spectrum analysis of *S. officinalis* extract with several biologically active moieties is depicted in Figure 2a. Multiple conjugate bonds and a phenol-based molecule of phytochemical composition were ascertained via two absorption peaks at 280 and 323 nm. *S. officinalis* aqueous extract with Ag^+^ and Cu^2+^ ions was determined at 433 and 572 nm wavelengths, respectively, in the UV spectra of biosynthesized Ag and Cu NPs. In addition, an Ag^+^ and Cu^2+^ ion combination was treated with an aqueous extract of *S. officinalis* in a biocompatible green synthesis of Ag-Cu NPs. The UV spectra reveal an absorption peak at a wavelength of 409 nm. However, in addition to the adsorption of biological moieties onto the surface of Ag-Cu nanoparticles, the observed wavelengths at 266 and 302 nm are functionality behind these phytochemical chemical moieties reflecting the successful biogenesis Ag-Cu nanoparticles. Meanwhile, the thickness of core–shell Cu also plays its role. It directly affects the absorption peak in UV-vis spectroscopy, which is investigated using localized surface plasmon resonance (LSPR) intensity, nanoparticle surface, structure effect, and UV-vis spectroscopy peak position [57,58]. Cu is a weak absorber at higher wavelengths. A strong LSPR peak in UV-vis spectroscopy is reflected at a comparatively lower wavelength (409 nm) in green-produced Ag-Cu nanoparticles. The obtained UV-vis spectrum demonstrates the effective synthesis/stabilization of Ag-Cu nanoparticles in the presence of various *S. officinalis* aqueous extract phytochemicals.

The functional groups of the phytomoieties present in the *S. officinalis* aqueous extract responsible for the reduction and stabilization of the metal ions of Ag and Cu were identified using Fourier transform infrared spectrometer analysis. *Salvia officinalis* aqueous extract possesses a variety of biomolecules, including rosmarinic acid, flavonoids, terpenes, steroids, tannins, glycosides, and saponins. The FTIR spectrum of *S. officinalis* aqueous extract illustrated in Figure 2b revealed peaks at 3338 cm^−1^, 2938 cm^−1^, 2853 cm^−1^, 1663 cm^−1^, 1464 cm^−1^, 1376 cm^−1^, 1282 cm^−1^, 1245 cm^−1^, 1154 cm^−1^, 1046 cm^−1^, 802 cm^−1^, and 705 cm^−1^. The FTIR spectrum of an oven-dried sample (uncalcined) of Ag-Cu NPs is shown in Figure 2b, which reveals the presence of bioactive molecules on the surface of Ag-Cu NPs [59]. The presence of prominent and identical peaks in both Ag-Cu NPs and *S. officinalis* aqueous extract demonstrates that the biologically active molecules can act as reducing and stabilizing agents during the Ag-Cu NPs’ formation [59]. The intense FTIR peak of uncalcined Ag-Cu NPs shown at 3335 cm^−1^ can be ascribed to phenolic O-H stretching and bending vibration. The peaks shown at wavenumbers 2944 cm^−1^ and 2866 cm^−1^ are assigned to C-H vibrations. The sharp peaks shown at 1694 cm^−1^ are due to the C=O stretching vibration of polyphenols. The peaks appearing at 1468 cm^−1^, 1376 cm^−1^, 1272 cm^−1^, 1046 cm^−1^, and 810 cm^−1^ are assigned to carboxylate group (COO) stretching vibration, CH bending, CO stretching, C-O stretching vibration, and C-H out-of-plane bending vibration, respectively. Figure 2b shows the FTIR spectrum of the calcined Ag-Cu NPs. The broad peaks at 3359 cm^−1^, 2933 cm^−1^, 1704 cm^−1^, and 1049 cm^−1^ are related to the strong O-H stretching vibration of a phenolic group, –C–H stretching vibration, C=O stretching vibration, and aromatic C–O stretching, respectively. The absorption bands at 1478 cm^−1^ and 1285 cm^−1^ are assigned to carboxylate group (COO) stretching vibration and CO stretching, respectively. The absorption band at 573 cm^−1^ might be due to the metal–oxygen bond. Meanwhile, in the *S. officinalis* extract, C–O bond stretching was observed after the presence of tertiary alcohol at 1157 cm^−1^; however, no such peak was observed in green-produced Ag-Cu NPs. In addition, the C–O stretching vibrations were seen at a comparatively lower frequency behind primary and secondary alcohols of *S. officinalis* moieties, as attributed to the band peak at 1051 cm^−1^. The overall functional group FTIR analysis revealed the successful biosynthesis of Ag-Cu NPs from an aqueous extract of *S. officinalis* (sage).

Raman spectroscopy studies were performed to obtain information about the surface-adsorbed functionalities. SERS is acknowledged as a helpful analytical approach for sensitive chemical analysis and interfacial studies, with numerous advantages, as a result of the ever-expanding research on plasmonic studies and the persistent enrichment of understanding. It is well-documented that molecules adsorbed on the rough surface of Ag-Cu NPs produce higher SERS signal intensities. Figure 2c shows the Raman spectrum of Ag-Cu NPs, which consist of vibrational modes at 230 cm^−1^, 432 cm^−1^, 588 cm^−1^, 654 cm^−1^, 703 cm^−1^, 1093 cm^−1^, 1453 cm^−1^, and 1625 cm^−1^. The Ag-Cu NPs were synthesized using *S. officinalis*, which acts as a reducing and stabilizing/capping agent for Ag-Cu NPs. The extract consisted of several organic constituents like polyphenols, flavonoids, tannins, proteins, carboxylic groups, and a hydroxyl group. The Raman band at 230 cm^−1^ is assigned to the frequency of the Ag-O bond. The vibrational peaks obtained from 432 cm^−1^−703 cm^−1^ arise from C-H bending vibration. The peak at 1093 cm^−1^ is attributed to aromatic C-O stretching vibration. The bands at 1453 cm^−1^ and 1625 cm^−1^ are obtained due to aromatic COO stretching vibration and C=O stretching vibration, respectively. The combined studies of FTIR and Raman spectra confirm that phytochemicals of the *Salvia officinalis* plant extract encapsulate the prepared NPs.

### 3.2. X-ray Diffraction Analysis

**Figure 3:**
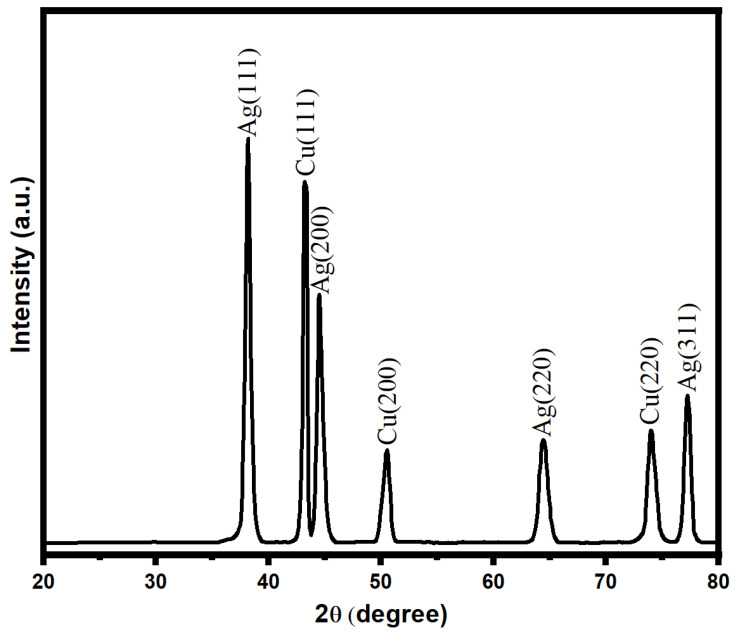
XRD pattern of bimetallic Ag-Cu nanoparticles.

The characterization of the bimetallic Ag-Cu nanoparticles was performed using the X-ray diffraction technique, as shown in Figure 3. The presence of discrete XRD patterns with respective diffraction peaks at 2θ was used to verify the crystalline nature of biogenic Ag-Cu nanoparticles. The existence of face-centered cubic (fcc) crystals of metallic silver was demonstrated by diffraction peaks recorded at angles (2θ) such as 38.5, 44.8, 64.7, and 77.5. However, these peaks were attained with corresponding crystallographic planes of (111), (200), (220), and (311), respectively. Due to the presence of copper, an XRD diffraction pattern with diffraction angles (2θ) at 43.8, 50.4, and 73.6 with respective crystal planes of copper nanoparticles of (111), (200), and (220) was observed. The XRD data that were obtained demonstrated unequivocally the presence of Cu nanoparticles with the fcc phase of Cu in conjunction with Ag NPs [60,61]. In addition, the Scherrer equation (d = K/cos) was used to determine the average crystallite size of the Ag-Cu NPs. It was calculated from this equation that the Ag-Cu NPs possess an average crystallite size of about 17 nm.

### 3.3. Surface Morphology Analysis

**Figure 4:**
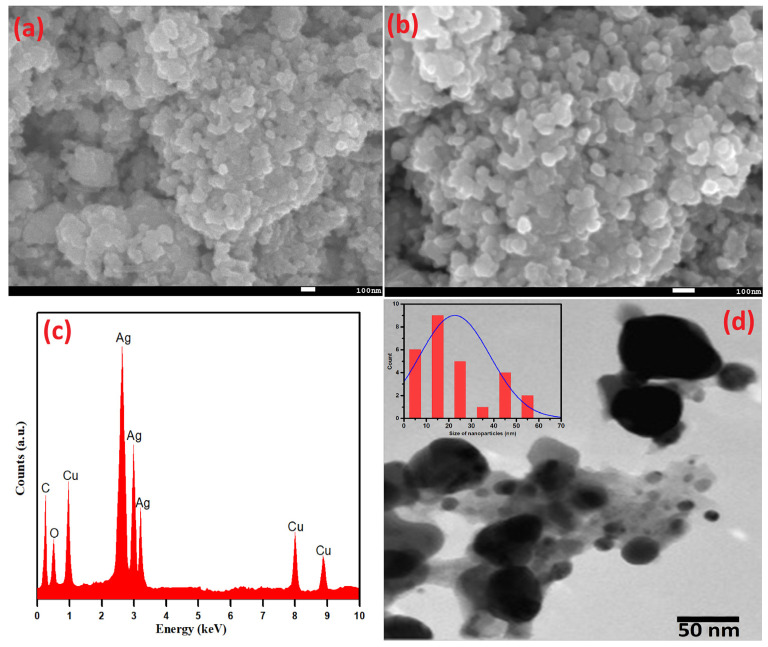
(**a**,**b**) SEM, (**c**) EDX, (**d**) TEM, and (insert) particle size distribution of Ag-Cu NPs.

Scanning electron microscopy (SEM) analysis was performed to deduce the surface morphology of Ag-Cu NPs. The as-prepared Ag-Cu NPs using *S. officinalis* aqueous extract biological moieties are spherical and evenly dispersed (Figure 4a,b). The SEM picture was taken at 15.0 kV with a 45 K resolution, and the chosen portion was retaken at a higher resolution of 70 K, as shown in Figure 4b. The uniform dispersion of nanoparticles is exhibited in both figures. As seen in Figure 4c, EDX analysis was used to estimate the presence of the metals Ag and Cu in a chosen area of the SEM image. The EDX examination further verified that the Ag-Cu NPs produced are made up of 47.83 wt% of Ag and 28.95 wt% of Cu. The presence of phytochemicals on the surface of the Ag-Cu NPs may be the cause of the signals for carbon and oxygen. The obtained results emphasize the comparatively higher peak intensity of Ag compared to Cu, indicating the crystalline nature of Ag-Cu NPs. Furthermore, it was confirmed that the biogenic moieties successfully stabilized Ag-Cu NPs while some EDX peaks for O and C were also seen at lower-energy sites.

Additionally, the shape and size of green synthetic Ag-Cu nanoparticles based on *Salvia officinalis* were determined by TEM, as shown in Figure 4d. With the specific particle coagulation and the difference in atomic radius of Cu^2+^ and Ag^+^ ions, different-sized nanoparticles, mostly Ag+ ions, were remarkably seen. These observations agree well with the EDX data. The peaks of Ag^+^ are more intense as compared to Cu^2+^ ions. An extensive size distribution of the detected nanoparticles (average particle size 22.6 nm) was estimated using the ImageJ imaging software and is displayed in Figure 4d (insert). The largest and smallest particle sizes were estimated to be 57.5 nm and 3.3 nm, respectively.

### 3.4. Minimum Inhibitory Concentration (MIC) of Ag-Cu NPs

Antimicrobial susceptibility testing of Ag-Cu NPs was performed by determining the MIC values of *Klebsiella pneumoniae*, *Escherichia coli*, *Staphylococcus aureus*, and *Staphylococcus epidermidis.* The MIC values demonstrated that Ag-Cu NPs inhibit the growth and survival of *Klebsiella pneumoniae*, *Escherichia coli*, *Staphylococcus aureus*, and *Staphylococcus epidermidis* at 10, 15, 5, and 5 µg/mL, respectively, as shown in Table 1. However, the positive control amoxicillin showed an MIC value of 2 µg/mL in all the bacterial strains. The lowest concentration of Ag-Cu NPs that allowed no visible growth of the bacterial strains was identified to be the MIC value.

Over the past few decades, antibiotic use and abuse, biofilms, and antibiotic resistance have dramatically increased. Thus, treating drug-resistant infections in clinics is complicated, even with new antibacterial drugs. To find a new drug with antibacterial properties, Ag-Cu NPs were synthesized. The MIC results showed that Ag-Cu NPs have potent antibacterial activity. The results follow previous studies in which synthesized ZnO NPs showed activity against an opportunistic pathogen, *Chromobacterium violaceum* [62].

The most promising possibilities to overcome antibiotic resistance are metal-based nanoparticles and their oxides. Silver oxide NPs, a type of silver nanoparticle, have been the subject of extensive research due to the antibacterial effects they are thought to have. Silver oxide NPs have little cytotoxicity to eukaryotic cells, show significant antibacterial and antifungal properties, and are relatively simple to produce [63].

### 3.5. Antibacterial Activity of Ag-Cu NPs by Agar Well Diffusion Assay

**Figure 5:**
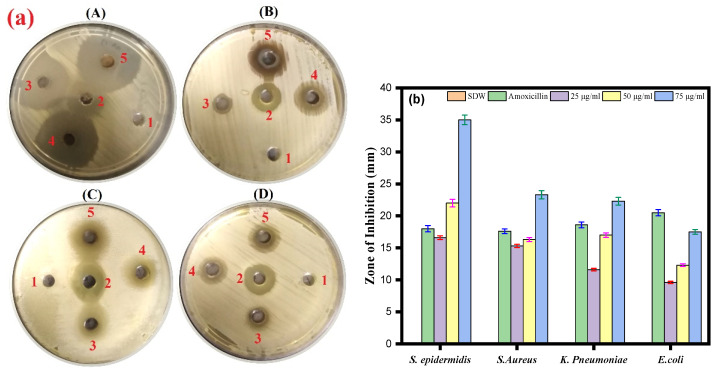
(**a**) Agar well diffusion assay. The plate represents the antibacterial activity of Ag-Cu NPs against four bacterial pathogenic strains and shows a clear zone (zone of inhibition in mm) produced by Ag-Cu NPs at different concentrations by well diffusion assay. 1 = negative control (SDW), 2 = amoxicillin (positive control), 3 = 25 µg/mL of Ag-Cu NPs, 4 = 50 µg/mL of Ag-Cu, and 5 = 75 µg/mL of Ag-Cu for (**A**) *S. epidermidis*, (**B**) *S. aureus*, (**C**) *E. Coli*, and (**D**) *K. pneumoniae*. (**b**) The bar diagram of the results of the zone of inhibition in mm at different concentration of Ag-Cu NPs obtained from the three different sets of experiments. Data are represented as mean ± SD of three different values.

The antibacterial activity of Ag-Cu NPs was further determined against Gram-negative bacterial strains *(Klebsiella pneumoniae* and *Escherichia coli*) and Gram-positive bacteria *(Staphylococcus aureus* and *Staphylococcus epidermidis*). The results of the agar well diffusion assay indicated that the inhibition zones range from a minimum of 9.6 ± 0.5 mm to a maximum of 35 ± 0.5 mm against all the bacterial strains (Figure 5a,b). The well diffusion method showed the highest antibacterial activity against *S. epidermidis* (35 mm), followed by *S. aureus* and *K. pneumoniae* (23.3 and 22.3 mm, respectively). In contrast, the lowest inhibition zone was against *E. coli* (17.3 mm) at a 75 µg/mL concentration for all assays. We also observed an effect of amoxicillin (17.6–20.3 mm) against bacterial pathogens.

It has been reported that silver and copper nanoparticles are effective antibacterial agents against *Staphylococcus aureus* and *Escherichia coli*, two of the most prevalent human pathogenic microorganisms. Furthermore, it was observed that antibacterial activity increased with the concentration of nanoparticles [64]. From our results of the well diffusion assay, we can see that synthesized Ag-Cu NPs can also inhibit the growth of *Staphylococcus aureus* and *Escherichia coli*.

### 3.6. Disk Diffusion Assay

**Figure 6:**
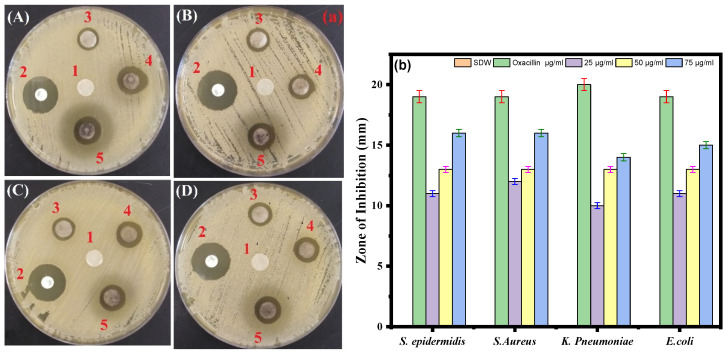
(**a**) Disc diffusion assay. The plate represents the antibacterial activity of Ag-Cu NPs against four bacterial pathogenic strains and shows a clear zone (zone of inhibition in mm) produced by Ag-Cu NPs at different concentrations by disc diffusion assay. 1 = negative control (SDW), 2 = oxacillin (positive control), 3 = 25 μg/mL of Ag-Cu NPs, 4 = 50 μg/mL of Ag-Cu, and 5 = 75 μg/mL of Ag-Cu for (**A**) *S. epidermidis*, (**B**) *S. aureus*, (**C**) *E. coli*, and (**D**) *K. pneumoniae*. (**b**) Bar diagram of the results of the zone of inhibition in mm at different concentrations of Ag-Cu NPs obtained from the three different sets of experiments. Data are represented as the mean ± SD of three different values.

Using the standard disk diffusion method, the antimicrobial effects of the biosynthesized Ag-Cu NPs against the four bacterial pathogens were tested. The *S. aureus* and *S. epidermidis* strains were most sensitive to biosynthesized Ag-Cu NPs at 75 µg/mL, with suppressive zones measuring 16 mm. In contrast, the *K. pneumoniae* strain was less sensitive to Ag-Cu NPS at 75 µg/mL, with a zone of inhibition of 14 mm. Oxacillin which is known to have a broad antibacterial effect, was used as a positive control (Figure 6a,b). Thus, our results are consistent with the previous study by Fan et al. in 2021 that demonstrates that Ag and Cu are effective antimicrobials, and the unique microstructures of synthetic Ag-Cu nanoalloys affect how well they perform. Furthermore, the antibacterial activity of Ag and Cu NPs is significantly boosted when combined, thus showing a synergistic effect [65]. In addition, studies showed that at micromolar concentrations, Ag-Cu NPs showed antibacterial activity against both Gram-positive and Gram-negative bacteria [66].

### 3.7. Biofilm Inhibition Activity

**Figure 7:**
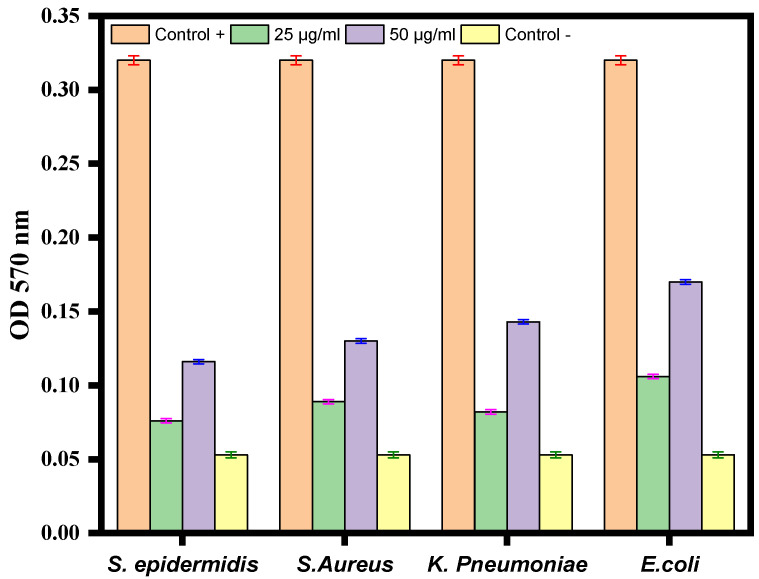
Antibiofilm activity of Ag-Cu NPS at 25 µg/mL and 50 µg/mL against *S. epidermidis*, *S. aureus*, *K. pneumoniae*, and *E. coli*. The negative control well was filled with medium only, and the positive control was medium with bacterial culture without any treatment. Data are represented as the mean ± SD of three different values.

Crystal violet staining was used to investigate the impact of Ag-Cu NPs on biofilm development. After being treated with varying quantities of Ag-Cu NPs, the ability of *S. epidermidis*, *S. aureus*, *K. pneumoniae*, and *E. coli* to build biofilms was altered (Figure 7). The results revealed a significant dose-dependent reduction of biofilm formation. In conclusion, treatment with Ag-Cu NPs inhibited biofilm development by 10–40%, with the most considerable inhibition observed at a dose of 50 µg/mL (Figure 7). Our results are consistent with our previous studies in which ZnO NPs altered biofilm formation by *C. violaceum* [62]. Biofilm inhibition by metallic nanoparticles has been reported in many studies. For example, Lange et al. found that silver and copper NPs could inhibit biofilm formation by mastitis pathogens [67].

## 4. Conclusions

In this research, stable Ag-Cu bimetallic nanoparticles with increased antibacterial characteristics were prepared from *S. officinalis* using a simple, environmentally friendly synthesis approach. The harm posed by the overuse and misuse of antibiotics has been related to the antibiotic resistance crisis. Ag-Cu NPs exhibit significant antibacterial action against *S. epidermidis*, *S. aureus*, *K. pneumococcus*, and *E. coli*. Our studies further demonstrated that Ag-Cu NPs possess substantial antibiofilm activity against four distinct bacterial pathogens. Overall, the data support Ag-Cu NPs as a possible target for future antibacterial medication discovery.

## Figures and Tables

**Table 1:** Minimum inhibitory concentration (MIC) of bacterial strains when treated with Ag-Cu NPs and Amoxicillin.

Strains	Ag-Cu NPs MIC (µg/mL)	Amoxicillin MIC (µg/mL)
*Klebsiella pneumoniae*	10	2
*Escherichia coli*	15	2
*Staphylococcus aureus*	5	2
*Staphylococcus epidermidis*	5	2

## Data Availability

All data created in this study are provided in this manuscript.
